# Inside Out: Archaeal Ectosymbionts Suggest a Second Model of Reduced-Genome Evolution

**DOI:** 10.3389/fmicb.2017.00384

**Published:** 2017-03-07

**Authors:** Trevor Nicks, Lilah Rahn-Lee

**Affiliations:** Department of Biology, William Jewell College, LibertyMO, USA

**Keywords:** nanoarchaea, ectosymbiont, endosymbiont, reduced-genome evolution, evolutionary biology, archaeal evolution

## Abstract

Reduced-genome symbionts and their organelle counterparts, which have even smaller genomes, are essential to the lives of many organisms. But how and why have these genomes become so small? Endosymbiotic genome reduction is a product of isolation within the host, followed by massive pseudogenization and gene loss often including DNA repair mechanisms. This phenomenon can be observed in insect endosymbionts such as the bacteria *Carsonella ruddii* and *Buchnera aphidicola*. Yet endosymbionts are not the only organisms with reduced genomes. Thermophilic microorganisms experience selective pressures that cause their genomes to become more compact and efficient. Nanoarchaea are thermophilic archaeal ectosymbionts that live on the surface of archaeal hosts. Their genomes, a full order of magnitude smaller than the *Escherichia coli* genome, are very small and efficient. How have the genomes of nanoarchaea and late-stage insect endosymbionts, which live in drastically different environments, come to mirror each other in both genome size and efficiency? Because of their growth at extreme temperatures and their exterior association with their host, nanoarchaea appear to have experienced genome reduction differently than mesophilic insect endosymbionts. We suggest that habitat-specific mechanisms of genome reduction result in fundamentally different pathways for these two groups of organisms. With this assertion, we propose two pathways of symbiosis-driven genome reduction; isolation-symbiosis experienced by insect endosymbionts and thermal-symbiosis experienced by nanoarchaea.

## Two Evolutionary Paths to a Reduced Genome

**FIGURE 1:**
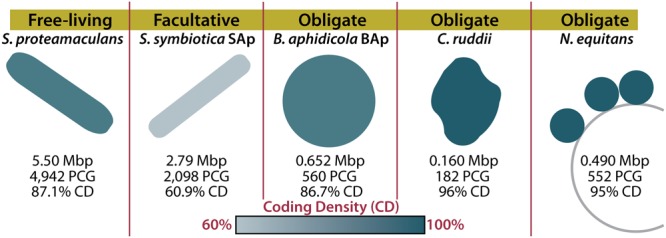
**Host dependency and symbiont genome structure in reduced-genome organisms.** Genome sizes and characteristics are shown for a free-living organism (*Serratia proteamaculans*), a facultative symbiont (*Serratia symbiotica* SAp), and three obligate symbionts (*Buchnera aphidicola* BAp, *Carsonella ruddii*, and *Nanoarchaeum equitans*). The color of the organism indicates the coding density of the genome. Mbp, mega base pairs; PCG, protein coding genes; CD, coding density (Waters et al., 2003; Nakabachi et al., 2006; Tamames et al., 2007; Lamelas et al., 2011).

In the depths of oceans and hot springs around the world live nanoarchaea. This archaeal phylum was first discovered in 2002, though only two members have been cultured and only a handful sequenced (Huber et al., 2002; Waters et al., 2003; Podar et al., 2013; Munson-McGee et al., 2015; Wurch et al., 2016). The cultured representatives, *Nanoarchaeum equitans* (0.490 Mbp) and *Nanopusillus acidilobi* (0.605 Mbp), are thermophilic obligate ectosymbionts with extremely reduced genomes and metabolic capacities, and as such can only be cultured in combination with their hosts. Like other nanoarchaea that have been imaged, they live on the external membranes of their archaeal hosts near aquatic thermal features (Waters et al., 2003; Stetter et al., 2009; Wurch et al., 2016, **Figure 1**). Although it is formally possible that these incredibly small genomes are an ancestral trait, the accepted theory is that nanoarchaea have experienced extensive gene loss (Waters et al., 2003; Podar et al., 2013), an experience that is strikingly similar to the gene loss experienced by another group of reduced-genome microbes with limited metabolic capacities: the bacterial endosymbionts of insects (0.140–0.706 Mbp). Insect endosymbionts have served as a model for how symbiosis drives genome reduction (Martínez-Cano et al., 2015). Nanoarchaea and long-term insect endosymbionts both have meager biosynthetic capabilities and few pseudogenes. However, the genomes of these two groups generally display three fundamental differences: nanoarchaea have retained their DNA repair mechanisms, and have reduced intergenic space and average gene length. Cumulatively, nanoarchaea have more efficient genomes than most insect endosymbionts, meaning they have a higher functional gene density. For example, the genome of *N. equitans* contains 552 coding sequences while the insect endosymbiont *Buchnera aphidicola* Sg (0.640 Mbp) contains only 545 (Tamas et al., 2002; Waters et al., 2003).

What caused these genomes to be small in different ways? Isolation within bacteriocytes typically initiates a process ending in obligate association for the formerly free-living organism. Because isolation impedes foreign DNA exposure, and thus horizontal gene transfer (HGT), it creates small populations susceptible to genetic drift. This fixes deleterious mutations, causing pseudogenziation and, with rare exceptions (Lopez-Madrigal et al., 2015), the loss of DNA repair and homologous recombination machinery (Moran, 1996; McCutcheon and Moran, 2012). Isolation within bacteriocytes prevents the genomic expansion and rescue that leads to large, complex genomes. For example, *Myxococcus xanthus* (9.14 Mbp) contains 1.4 Mbp of genetic material gained through HGT (Goldman et al., 2004). With a plethora of sensory and regulatory genes, *M. xanthus* creates multi-cellular structures, differentiates individual cells within those structures, and hunts in groups. In contrast, endosymbionts have few functions besides informational processes and production of metabolites necessary for their host’s survival (McCutcheon and Moran, 2012).

Nanoarchaea are different. Because they are located on the outside surface of their hosts, nanoarchaea have access to foreign DNA, so isolation cannot explain genome loss in these organisms. In fact, three lines of evidence suggest that genome reduction should not occur in nanoarchaea. (1) There is evidence for nanoarchaeal genetic material acquisition from viruses (Munson-McGee et al., 2015). Additionally, nanoarchaea are theoretically capable of undergoing HGT because they (2) retain homologous recombination and DNA repair mechanisms and (3) are ectosymbionts and thus are not as susceptible to drift as endosymbionts (Waters et al., 2003; Wurch et al., 2016). To understand why nanoarchaea and insect endosymbionts have similarly small genomes, let’s consider each group separately.

## The Lonely Road: Isolation and Loss

**FIGURE 2:**
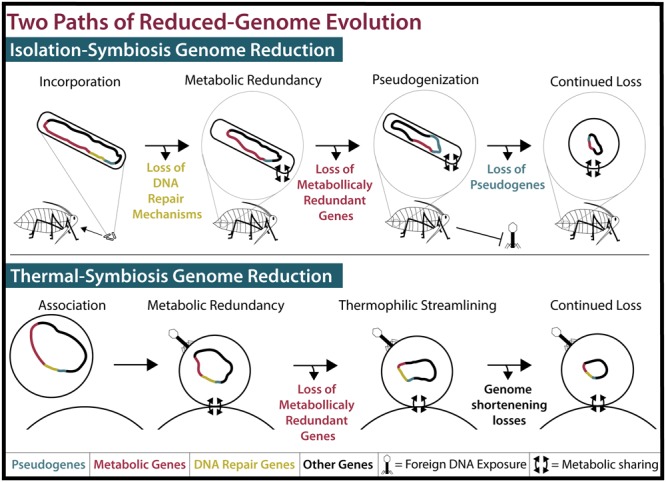
**Two models of reduced-genome evolution. (Top)** Isolation-symbiosis genome reduction begins with the incorporation of a free-living bacteria into the bacteriocytes of an insect, inhibiting foreign DNA exposure. Host-microbe metabolite sharing renders many metabolic genes (Red) redundant. Deleterious mutations in DNA repair mechanisms (Yellow) and their loss cause genome-wide pseudogenization (Blue) and pseudogene loss. While displayed stepwise, loss of pseudogenes and metabolically redundant genes concurrently create tiny efficient genomes. As this process progresses, cell shape determining genes are lost and endosymbiont cells become round or pleiotropic (McCutcheon and Moran, 2012). **(Bottom**) Thermal-symbiosis genome reduction begins with the association of two streamlined thermophilic archaea. Foreign DNA exposure occurs and DNA repair mechanisms are maintained. Concurrently, metabolically redundant genes and continued thermophilic streamlining create small efficient genomes.

When considering how an organism evolved, it’s important to consider what demands are enforced by the environment. Many insects rely on their endosymbionts for the production of metabolites such as amino acids that they cannot synthesize or gain from their diets (McCutcheon and Moran, 2012). Ancient insect endosymbionts are characterized by reduced metabolic capabilities, reduced DNA repair and recombination genes, and reduced pseudogenes (McCutcheon and Moran, 2012). These characteristics can be attributed to a four-step process of genome reduction, resulting from their isolation (**Figure 2**; for a detailed review, see McCutcheon and Moran, 2012). The first step in insect endosymbiont genome reduction is the incorporation of an independent bacterium into an insect, resulting in that bacteria’s isolation within the host. Endosymbionts then live in small populations and are maternally transferred to future insect generations (Moran, 1996). The second and third steps begin immediately upon incorporation into the host and occur concurrently. The second step of genome reduction is caused by the buffet of metabolites such as nucleotides and lipids that endosymbionts receive from their hosts (van Ham et al., 2003; Moran and Bennett, 2014). As long as an endosymbiont receives sufficient amounts of these products, its own biosynthetic genes become superfluous or metabolically redundant and thus prone to loss via genetic drift. Third, the isolation experienced by insect endosymbionts renders them incapable of receiving external genetic material that would normally restore functions lost by mutation. Eventually, the genes encoding DNA repair machinery may suffer irreparable mutations. In sum, this leads to genetic drift and pseudogenization in non-essential genes, which is exacerbated by the loss of DNA repair mechanisms. The accumulation of pseudogenes and metabolic redundancy results in the fourth step: continued gene loss (McCutcheon and Moran, 2012). All organisms have an innate bias toward deletion (Mira et al., 2001). This affinity for gene loss in insect endosymbionts is likely compounded by the loss of repair and recombination mechanisms. Eventually, most pseudogenes, non-essential metabolic genes, and DNA repair genes are lost in insect endosymbionts, resulting in a tiny efficient genome (McCutcheon and Moran, 2012). It is possible to witness the intermediate stages of this isolation-driven genome reduction in insect endosymbionts living today (Manzano-Marín and Latorre, 2016; **Figure 2**). Examples include the co-symbionts of the aphid *Acyrthosiphon pisum*: the recently incorporated *Serratia symbiotica* SAp (2.79 Mbp) and the ancient endosymbiont, *B. aphidicola* BAp (0.652 Mbp) (Lamelas et al., 2011; **Figure 1**). SAp is at the pseudogene proliferation step of genome reduction. It has 550 pseudogenes and a coding density of 60.9%, while its free-living relative, *Serratia proteamaculans* (5.50 Mbp), has 12 pseudogenes and a coding density of 87.1% (Burke and Moran, 2011). As the insect endosymbiont pathway of reduced-genome evolution would suggest, deletional bias and mutation has removed the majority of pseudogenes, non-essential biosynthetic genes, and DNA repair genes in the small-genomed BAp, which has only 12 pseudogenes and a coding density of 86.7% (Lamelas et al., 2011). BAp is utterly reliant on its host for the production of lipids and nucleotides. Other strains of *B. aphidicola* have even lost amino acid pathways and rely on co-symbionts for tryptophan production (Pérez-Brocal et al., 2006; Lamelas et al., 2011). The tiny genome of the psyllid endosymbiont *Carsonella ruddii* (0.156 Mbp), which does not even encode enough protein machinery to replicate, shows how this process can result in a genome that more closely resembles an organelle than an organism (Tamames et al., 2007). Thus, isolation limits foreign DNA exposure (Moran, 1996; Sloan et al., 2014) and creates an environment where pseudogenization and gene loss occur. Furthermore, metabolic redundancy caused by sharing of metabolites and a natural inclination for deletion lead to the loss of redundant metabolic genes and the evolution of reduced genomes in endosymbionts.

## Lean On Me: Host Reliance and Genome Reduction

The second stage of genome reduction in insect endosymbionts, as stated above, is caused by the redundancy of biosynthetic genes whose products are also provided by the host. Similar metabolite sharing in nanoarchaea may explain their lack of metabolic genes, as nanoarchaea produce almost nothing for themselves. Both of the cultured nanoarchaea lack nearly all genes necessary for the production of lipids, nucleotides, cofactors, and amino acids (Waters et al., 2003; Wurch et al., 2016). Presumably, these metabolites and even ATP are shared with nanoarchaea from their hosts via unknown mechanisms (Giannone et al., 2014). *N. equitans* also lacks genes necessary for glycolysis and other carbon cycling pathways (Waters et al., 2003). However, *N. acidilobi*, with its slightly larger genome, has retained enzymes responsible for carbohydrate metabolism (Wurch et al., 2016). If host biosynthesis of metabolites is a driving factor in the genome reduction of nanoarchaea, the retention of some metabolic enzymes and the larger genome of *N. acidilobi* may indicate that *N. equitans* is further along in this process than *N. acidilobi.* We propose that, as for insect endosymbionts, the sharing of metabolites between nanoarchaea and their hosts puts the biosynthetic genes of nanoarchaea at risk of deletion. The importance of metabolic redundancy in both insect endosymbiont and nanoarchaeal genome reduction suggests that a universal requirement of extreme genome reduction may be the sharing of metabolites between two organisms.

## The Outsiders: Interactions With Foreign Genetic Material

However, not all features of the insect endosymbiont model apply to nanoarchaea. As ectosymbionts, nanoarchaea do not experience isolation from foreign DNA like insect endosymbionts (**Figure 2**). This is evidenced by Nanoarchaeal uptake of foreign DNA. Genome fragments of *Nanobsidianus*, an uncultured nanoarchaean from a Yellowstone National Park hot spring, have been shown to contain viral genes. Viral fractions from the same hot spring contained matching viral DNA, suggesting that *Nanobsidianus* species support viral replication (Munson-McGee et al., 2015). Although viral DNA transfer has not been documented in other nanoarchaea, both of the cultured nanoarchaea possess a full arsenal of DNA repair genes, and an array of genes indicating they are capable of homologous recombination (Waters et al., 2003; Wurch et al., 2016) and presumably HGT. Therefore, it is unlikely that genome reduction in nanoarchaea is the result of pseudogenization and subsequent loss.

## Give It, Lose It, Take It: Gene Sharing Between Symbiont and Host

Even though isolation limits endosymbiont exposure to foreign DNA, HGT still affects endosymbiont evolution by passing genes to and creating genetic redundancy with their hosts. For example, the pea aphid *A. pisum* expresses peptidoglycan synthesis genes in its bacteriocytes that likely compensate for genes missing from its endosymbiont (Nikoh et al., 2010). Similarly, there is evidence for HGT between nanoarchaea and their hosts. Thirteen genes have been identified for which the versions carried by *N. equitans* and by its host *Ignicoccus hospitalis* (1.29 Mbp) share the highest sequence similarity (Podar et al., 2008). It could be that genes from *N. equitans* have been transferred to the genome of *I. hospitalis*. If the products of these homologous genes are functionally equivalent in *I. hospitalis* and *N. equitans*, one copy may be at risk for loss. Genes already lost from *N. equitans* in this manner would be hard to identify, but a comparison of homologs, if any, between *I. hospitalis* and *N. equitans*’ less-reduced relative *N. acidilobi* may indicate if transfer of genes to the host is a major player in nanoarcheal genome reduction.

## It’S Getting Hot In Here: Thermal Selective Pressures

So far we have considered how the forces driving genome reduction in nanoarchaea and insect endosymbionts differ due to their ectosymbiotic and endosymbiotic lifestyles. If nanoarchaea are not isolated genetically, what other factors contribute to their genome reduction? Nanoarchaea and insect endosymbionts differ in their thermophilic and mesophilic lifestyles. Compared to those of mesophiles, thermophilic genomes are generally characterized by shorter genes, reduced intergenic space, and decreased non-synonymous mutations (Sabath et al., 2013; Wang et al., 2015). This raises the questions: how and why do thermophiles reduce their genomes, and to what extent has this influenced genome reduction in nanoarchaea?

It was recently reported that thermophilic bacteria demonstrate a negative correlation between growth temperature and genome size (Sabath et al., 2013). This correlation, while present, is not significant for free-living thermophilic archaea. Whether this indicates a fundamental difference in mechanisms of thermal selection on archaea compared with bacteria, or whether this is due to the limited availability of diverse archaeal genome sequences remains to be seen. In the case of bacteria, however, there is evidence that this is more than a correlation: small genomes are adaptive at high temperatures.

By selecting for thermotolerant *Escherichia coli* Blaby et al. (2012), showed that genes are lost as mesophiles evolve thermotolerance. In this laboratory evolution experiment, *E. coli* were cultured over eight months with temperatures increasing from 44 to 49.7°C. Why would high growth temperatures favor gene loss? As in bacteria, genome reduction in thermophilic archaea may be a result of energetic stress minimization and genome streamlining (Valentine, 2007; Martínez-Cano et al., 2015; Wang et al., 2015). Decreased genome size may allow for a reduction in overall cell size, decreasing the energy required for cell maintenance (Wang et al., 2015), and selecting for the loss of genes providing little to no benefit. In the case of Blaby et al. (2012), one evolved thermotolerant strain lost the glyceroporin gene (*glpF*), which encodes a glycerol importer. Associated with this loss was an increase in the optimal growing temperature of Δ*glpF*, perhaps due to increased membrane stability in the absence of the transporter. Presumably, the loss of *glpF* renders the entire pathway for glycerol catabolism useless, and further experimental evolution would lead to the loss of the rest of the pathway.

In addition to having fewer genes, thermophiles have genes that are often shorter than their mesophilic homologs. Structure-destabilizing loops are lost in the proteins of thermophiles, which must retain thermodynamic stability at high temperatures (Thompson and Eisenberg, 1999; Wang et al., 2015). For instance, the CheY response regulator of *Thermotoga maritima* has a shorter surface loop compared to its homolog in *E. coli* that makes it more thermodynamically stable (Usher et al., 1998). Nanoarchaea have a reduced average gene length in comparison to most prokaryotes (Waters et al., 2003; Xu et al., 2005), perhaps because of thermophilic stabilizing losses. Interestingly, there is also evidence for similar losses in the proteomes of some insect endosymbionts (Manzano-Marín and Latorre, 2016). These losses may be a cellular economization to reduce energy usage, either because organisms are living in energy-poor environments like hydrothermal vents, or to reduce the metabolic load on their hosts. Unlike insect endosymbionts, nanoarchaea are in both positions and may experience gene-shortening pressures as both symbionts and thermophiles.

Temperature-driven selection for smaller cells may also play a role in reducing the genome size of nanoarchaea. Decreased cell diameters can be advantageous for thermophilic organisms as maintenance is minimized and surface-to-volume ratios are optimized for nutrient uptake. Sabath et al. (2013) have shown that cell size can be a function of genome size, and that pressures driving size reduction may play a role in reducing genome size in *Pelagibacter ubique* (1.31 Mbp), which has one of the smallest volumes of free-living organisms (Giovannoni et al., 2005). Incredibly, the cells of *N. acidilobi* can be as small as 100 μm in diameter (Wurch et al., 2016). The small genomes of nanoarchaea may be a result of selective pressures favoring smaller cells, a trait that would allow nanoarchaea to be less of an energetic burden to their host in energy poor thermophilic environments.

As described above, thermophiles experience pressures resulting in fewer genes, shorter genes, and small cell sizes. These pressures may contribute not only to genome reduction in nanoarchaea but also to the efficient character of their genomes. Although DNA is less stable at higher temperatures, mutations are fixed less often in thermophiles than in mesophiles (Beeby et al., 2005). Thermophiles have fewer non-synonymous mutations than mesophiles due to sequence-retention constraint, as even minor alterations in thermophilic proteomes can be deadly (Wang et al., 2015). This pressure likely plays a role in the retention of nanoarchaeal DNA repair mechanisms, which help nanoarchaea avoid pseudogenization. Living in extreme environments has clearly shaped the small yet efficient genomes of nanoarchaea.

## Small Beginnings: A Second Model of Reduced-Genome Evolution

In conclusion, we suggest that nanoarchaea and insect endosymbionts achieved their reduced genomes through two different pathways (**Figure 2**). Nanoarchaeal genome reduction, as we propose, is the result of two thermophilic archaea becoming membrane-associated. This association leads to the sharing of metabolites from a host, such as *I. hospitalis*, to an ectosymbiont, such as *N. equitans*, leading to metabolic redundancy and the subsequent loss of the ectosymbiont’s biosynthetic genes. This genome shortening is exacerbated by the pressures of a thermophilic lifestyle, leading to reduced average gene length, a decrease in intergenic space, and a tiny cell size. We term this “thermal-symbiosis genome reduction.” With the emergence of evidence supporting a second model of symbiosis-driven reduced-genome evolution, we propose that the pathway previously summarized by McCutcheon and Moran (2012) be referred to as “isolation-symbiosis genome reduction.” It is important to note that isolation-symbiosis genome reduction accounts for the reduction of a mesophilic bacterial genome of any initial size down to that of the miniscule *C. ruddii*. For example, the range of *S. symbiotica* genome sizes (0.65–3.58 Mbp) and coding densities (39–79%) in strains isolated within different host species evidences how the genome of a once free-living organism can erode (Manzano-Marín and Latorre, 2016). However, thermal-symbiosis relies not on isolation and pseudogenization but metabolic redundancy and adaptation. Furthermore, archaea, which may have ancestrally smaller genomes (Sabath et al., 2013), have less to lose during symbiosis. If the last free-living ancestor of the cultured nanoarchaea was thermophilic then its genome likely had economized features. Future research, including the culturing and sequencing of more thermophilic nanoarchaeal species, may point toward the last free-living ancestor of nanoarchaea being a small-genomed, efficient thermophile like the free-living *I. hospitalis*.

Nanoarchaea were originally characterized as thermophiles, but improved 16S primers have since led to their identification (Casanueva et al., 2008; Stetter et al., 2009), though not their observation, isolation, or sequencing, from mesophilic and halophilic environments. These organisms, from salt lakes of South Africa and Mongolia, will have experienced very different selective pressure than *N. equitans* and *N. acidilobi*. The thermal-symbiosis model presented above is specific to thermophiles and organisms in other energy poor environments. It will be especially interesting to see what culturing and sequencing of mesophilic, halophilic nanoarchaea will yield. Do they have reduced metabolic capabilities like *N. acidilobi* and *N. equitans*? What size are their genomes? Are they even symbiotic? More genomic data from a diverse range of nanoarchaea will address these and other questions surrounding the roles of symbiosis and temperature in the genome reduction of bacteria and archaea.

## Author Contribution

TN and LR-L conceived, wrote, and edited the review.

## Conflict of Interest Statement

The authors declare that the research was conducted in the absence of any commercial or financial relationships that could be construed as a potential conflict of interest.
